# Patient Portals to Support Care Partner Engagement in Adolescent and Adult Populations: A Scoping Review

**DOI:** 10.1001/jamanetworkopen.2022.48696

**Published:** 2022-12-28

**Authors:** Kelly T. Gleason, Danielle Peereboom, Aleksandra Wec, Jennifer L. Wolff

**Affiliations:** 1 Johns Hopkins University School of Nursing, Baltimore, Maryland; 2 Johns Hopkins Bloomberg School of Public Health, Baltimore, Maryland

## Abstract

**Importance:**

Family and other unpaid care partners may bridge accessibility challenges in interacting with the patient portal, but the extent and nature of this involvement is not well understood.

**Objective:**

To inform an emerging research agenda directed at more purposeful inclusion of care partners within the context of digital health equity by (1) quantifying care partners’ uptake and use of the patient portal in adolescent and adult patients, (2) identifying factors involving care partners’ portal use across domains of the System Engineering Initiative for Patient Safety model, and (3) assessing evidence of perceived or actual outcomes of care partners’ portal use.

**Evidence Review:**

Following Arksey and O’Malley’s methodologic framework, a scoping review of manuscripts published February 1 and March 22, 2022, was conducted by hand and a systematic search of PubMed, PsycInfo, Embase, and Web of Science. The search yielded 278 articles; 125 were selected for full-text review and 41 were included.

**Findings:**

Few adult patient portal accounts had 1 or more formally registered care partners (<3% in 7 of 7 articles), but care partners commonly used the portal (8 of 13 contributing articles reported >30% use). Care partners less often authored portal messages with their own identity credentials (<3% of portal messages in 3 of 3 articles) than with patient credentials (20%-60% of portal messages in 3 of 5 articles). Facilitators of care partner portal use included markers of patient vulnerability (13 articles), care partner characteristics (15 articles; being female, family, and competent in health system navigation), and task-based factors pertaining to ease of information access and care coordination. Environmental (26 articles) and process factors (19 articles, eg, organizational portal registration procedures, protection of privacy, and functionality) were identified as influential to care partner portal use, but findings were nuanced and precluded reporting on effects. Care partner portal use was identified as contributing to both patient and care partner insight into patient health (9 articles), activation (7 articles), continuity of care (8 articles), and convenience (6 articles).

**Conclusions and Relevance:**

In this scoping review, care partners were found to be infrequently registered for the patient portal and more often engaged in portal use with patient identity credentials. Formally registering care partners for the portal was identified as conferring potential benefits for patients, care partners, and care quality.

## Key Points

**Question:**

What is known about the frequency, nature, and consequences of care partners’ uptake and use of the patient portal?

**Findings:**

This scoping review including 41 studies noted that few care partners are formally registered for the patient portal but that informal portal use, with patient identity credentials, is common. Patients and care partners identified the need for and potential benefits of care partner engagement in the patient portal.

**Meaning:**

The findings of this scoping review suggest that health care systems have directed limited effort toward engaging care partners in the patient portal, despite identified potential benefits.

## Teaser

This scoping review describes the use of patient portals by patient care partners.

## Introduction

The patient portal has assumed a prominent role in efforts to engage patients in their care through facilitating information access, health care management, and communication with the care team.^1,2,3,4,5,6^ Systematic reviews suggest that use of the patient portal may be associated with improved patient decision-making, self-efficacy, and behavioral outcomes, such as treatment adherence,^7,8,9^ through varied pathways involving convenience, continuity in care, activation, and understanding.^10,11,12^ However, individuals who are older, less educated, and with less technology experience are less likely to use a patient portal,^13,14^ less able to perform health management tasks electronically,^15,16^ and more reliant on others to navigate health system demands.^17^

Care partner engagement may bridge digital literacy demands of patients who have difficulty accessing the patient portal.^18,19^ Many health systems allow patients to authorize a family member or trusted friend shared access to their patient portal account using the designee’s own identity credentials.^20^ Preliminary studies suggest that shared access may facilitate improved patient and family satisfaction with communication, agreement about treatments, and confidence in managing care^21,22^ and that it is commonly desired by patients and valued by families.^23^

To our knowledge, national information enumerating uptake of shared access does not exist, but implementation of this functionality by health systems has been variable,^20,24^ and anecdotal evidence suggests it is limited.^25,26^ In the absence of straightforward processes to access patient health information with their own credentials, care partners may access the portal using patient credentials.^7,26,27^ Having a stronger understanding of the extent and nature of care partners’ portal use could benefit efforts to support safe and clinically appropriate care that is respectful of and responsive to patient preferences and promote learning health system initiatives^28,29^ to monitor care quality and outcomes in populations with greater vulnerability.^30,31^

Consistent with the nascent and diffuse nature of available evidence, we undertook a scoping review to consolidate studies that have examined care partner use of patient portals in adolescent and adult patient populations. We had 3 aims: (1) to identify studies that have quantified the extent of care partner uptake and use of the patient portal; (2) to assess the extent and type of evidence identifying factors affecting care partner use of the patient portal, drawing on the System Engineering Initiative for Patient Safety (SEIPS) model; and (3) to map findings from studies that have examined perceived or actual effects of care partner use of the patient portal on care partner capacity and patient outcomes. The overall objective of our study was to inform an emerging research agenda directed at more purposeful inclusion of care partners within the context of digital health equity.

## Methods

Our methods align with the scoping review typology in supporting systematic, transparent, and replicable results and consolidating evidence that pertains to an emerging topic of inquiry.^32,33^ We followed Arksey and O’Malley’s^34^ methodologic framework for scoping reviews and the Preferred Reporting Items for Systematic Reviews and Meta-analyses (PRISMA) extension for a scoping review checklist. Our definition of care partners encompasses nonclinicians other than the patient who access or use the patient portal on the patient’s behalf, including family members, trusted friends, and legal guardians. We defined the patient portal as a patient-facing, web-based technology offered by health care organizations to provide patients with access to their medical records.

### Study Identification

**Figure 1.**
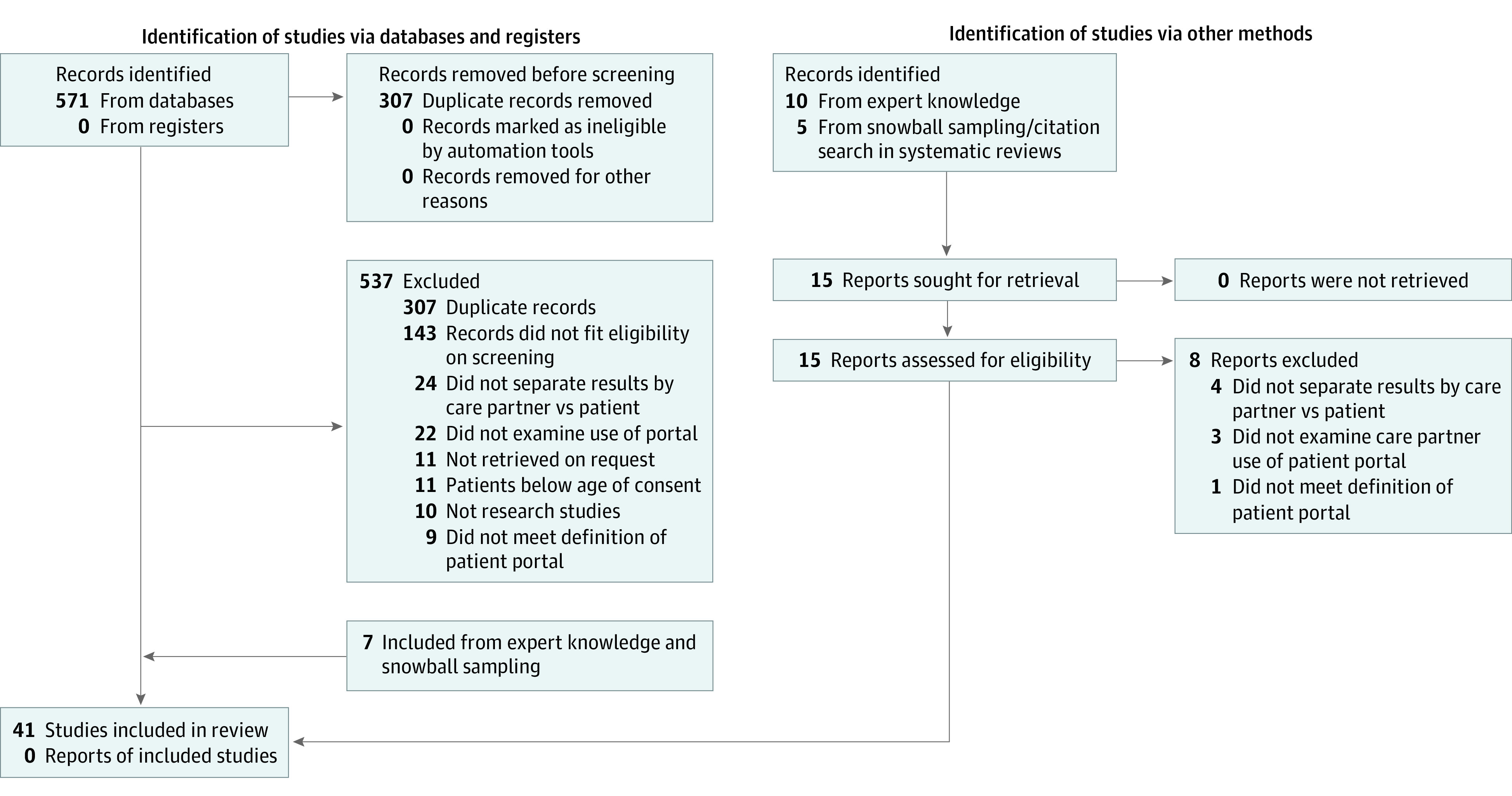
Preferred Reporting Items for Systematic Reviews and Meta-analyses Flow Diagram of Scoping Review Search

We iteratively developed the search strategy in consultation with an experienced medical librarian (eTable 1 in Supplement 1). We searched PubMed, Web of Science, Embase, and PsycInfo using search terms and MeSH headings that included key words related to *patient portal* in combination with *care partner*, *(informal) caregiver*, *family*, *parent*, *proxy*, and *legal guardian*. We conducted our search between February 1 and March 2, 2022. No date, language, or location limits were imposed. We included peer-reviewed qualitative and quantitative articles describing care partner use of the patient portal. We additionally conducted a hand search of the literature using expert knowledge and drawing on reference lists of relevant literature reviews. Reviews and nonempirical articles, such as commentaries, and articles using the term *caregiver* but describing professional health care professionals or clinicians were excluded. Articles were excluded that did not meet our definition of a patient portal or did not separate care partner use from patient use of the portal. We excluded studies of pediatric populations under the age of consent due to the well-accepted legal authority of parents and guardians. The PRISMA diagram (Figure 1) summarizes our search and screening process.

### Article Selection

Our 2-step screening process, facilitated by Covidence 2022 literature review software, consisted of title and abstract scan, followed by full-text review by 2 independent screeners (K.T.G., D.P., and A.W.) to confirm article eligibility. During the full-text review, reviewers applied inclusion and exclusion criteria to the remaining articles and discussed discrepancies as a group to reach consensus.

### Charting the Data and Collating, Summarizing, and Reporting Results

A data extraction tool from the Joanna Briggs Institute^33^ was adapted (eTable 2 in Supplement 1). The approach to summarizing and reporting on findings from identified articles varied by research question (RQ). We first compiled articles that quantitatively assessed care partners’ uptake and use of patient portals (RQ1) by focusing on key parameters, such as year, setting, population, and identity credentials (patient vs care partner), including use of direct messaging,^18,35^ which is uniquely able to determine sender identity. We then performed a thematic analysis to synthesize information from articles that reported on factors associated with care partner engagement (RQ2) and outcomes resulting from care partner portal use (RQ3). Analyses pertaining to RQ2 involved mapping each factor to the components described in the SEIPS model^36,37^ and categorizing findings by whether each factor was a facilitator, barrier, nonsignificant (quantitative analyses), or did not specify directionality with respect to motivating care partner uptake and use of the patient portal. Analyses pertaining to RQ3 involved mapping findings to consequences of care partner use of the portal following the Otte-Trojel framework,^11^ which we adapted to reflect care partner involvement.

## Results

Characteristics of included articles are summarized in Table 1 and individually presented in eTable 3 in Supplement 1. Of the 41 articles included in this review, 24 used quantitative methods, 10 used qualitative methods, and 7 used mixed methods. All but 4 studies were conducted in the US, and nearly half (19 [46.3%]) were published in 2020-2022. Most of the studies examined adults (23 [56.1%]); fewer studies examined adolescents (7 [17.1%]) or older adults (5 [12.2%]).

**Table 1.** Characteristics of 41 Included Studies

Variable	Studies, No. (%)			
	RQ1^a^	RQ2^b^	RQ3^c^	Total
Country				
US	18 (100)	20 (83.3)	12 (92.3)	37 (90.2)
Other^d^	0	4 (16.7)	1 (7.7)	4 (9.8)
Year published				
2020-2022	10 (55.6)	11 (45.8)	6 (46.2)	19 (46.3)
2015-2019	7 (38.9)	10 (41.7)	6 (46.2)	17 (41.5)
2014 or earlier	1 (5.6)	3 (12.5)	1 (7.7)	5 (12.2)
Data collection				
Focus groups/interviews	0	10 (41.7)	6 (46.2)	10 (24.4)
Survey	5 (27.8)	8 (33.3)	1 (7.7)	12 (29.3)
EMR/portal data	11 (61.1)	3 (12.5)	2 (15.4)	13 (31.7)
Multiple	2 (11.1)	3 (12.5)	4 (30.8)	6 (14.6)
Approach				
Quantitative	15 (83.3)	11 (45.8)	4 (30.8)	24 (58.5)
Qualitative	0	10 (41.7)	6 (46.2)	10 (24.4)
Mixed methods	3 (16.7)	3 (12.5)	3 (23.1)	7 (17.1)
Setting				
Health system	11 (61.1)	11 (45.8)	6 (46.2)	17 (41.5)
Multiple health systems	1 (5.6)	6 (25.0)	2 (15.4)	8 (19.5)
Clinical practice site	2 (11.1)	8 (33.3)	3 (23.1)	10 (24.4)
Outside health care delivery	4 (22.2)	4 (16.7)	2 (15.4)	6 (14.6)
Sample size				
<50	0	8 (33.3)	4 (30.8)	8 (19.5)
50-99	0	5 (20.8)	3 (23.1)	6 (14.6)
100-499	4 (22.2)	8 (33.3)	3 (23.1)	9 (22.0)
≥500	14 (77.8)	3 (12.5)	3 (23.1)	18 (43.9)
Age group				
Adolescents	4 (22.2)	2 (8.3)	1 (7.7)	7 (17.1)
Adults	12 (66.7)	12 (50.0)	7 (53.8)	23 (56.1)
Older adults	1 (5.6)	5 (20.8)	4 (30.8)	5 (12.2)
All ages	1 (5.6)	2 (8.3)	1 (7.7)	3 (7.3)
NA or unknown	0	3 (12.5)	0	3 (7.3)
Sample characteristics				
General patient population	12 (66.7)	11 (45.8)	7 (53.8)	24 (58.5)
Illness specific	6 (33.3)	13 (54.2)	6 (46.2)	17 (41.5)

Abbreviations: EMR, electronic medical record; NA, not available; RQ, research question.

^a^ What is the extent and nature of evidence quantifying care partners’ use of the patient portal in adolescent and adult patient populations, and to what extent does available evidence differentiate between the use of care partner vs patient identity credentials?

^b^ To what extent has prior evidence identified people, environmental, tools, and task-specific factors drawn from the System Engineering Initiative for Patient Safety model, affecting care partner access and use of the patient portal?

^c^ What is the extent of available evidence describing perceived or actual effects of care partner use of the patient portal on care partner capacity (knowledge and behaviors) and patient outcomes?

^d^ Two studies were conducted in Canada, 1 in Germany, and 1 in Ireland.

### Care Partner Registration and Use of the Patient Portal

Seven articles described care partner registration for adolescent (n = 1) or adult (n = 6) portal accounts from date/time-stamped electronic health record data (Table 2). Analyses were primarily conducted at academic medical centers (n = 4) or integrated managed care consortiums (n = 2) and about evenly divided between examining all registered users (n = 4) or cohorts of disease-specific populations (n = 3). All 6 studies of adult patients found less than 3% of portal accounts to have a formally registered care partner. The 1 study of adolescent patients reported 25.7% of portal accounts belonged to the adolescent, 0.8% belonged to a delegate (eg, parent or guardian of an adult for adolescents aged ≥18), and 73.5% belonged to a surrogate (eg, parent or guardian of a child).

**Table 2.** Articles Examining Care Partner Access and Use of the Patient Portal

Study characteristic	Adolescents	Adults
**Panel A: care partner registration for the portal**		
No. of articles	1	6
Health care professional		
Academic medical center	1	3
Integrated managed care consortium	0	2
Not reported	0	1
Inclusion criteria		
Registered users	1	3
Cohort defined by health condition	0	3
Care partner registration, %		
<1	1^a^	1
1-3	0	5
>3	1^a^	0
**Panel B: care partner use of the portal**		
No. of articles	3	10
Data source		
Electronic medical record	3	4
Survey	0	6
Health care professional		
Academic medical center	3	3
Integrated managed care consortium	0	2
Not reported	0	5
Proportion of care partners using the portal, %		
<10	0	2
10-30	0	3
>30	3	5
**Panel C: care partner engagement in direct messaging through the portal**		
No. of articles	1	5
Health care professional		
Academic medical center	0	2
Integrated managed care consortium	0	1
Other/not reported	1	2
Inclusion criteria		
Registered users	1	3
Cohort defined by health condition	0	2
Care partner direct messaging		
Using own identity credentials, %		
<1	0	1
1-3	0	2
Using patient identity credentials, %		
<5	0	1
5-20	0	1
21-60	1	2

^a^ Steitz et al^38^ differentiated delegates from surrogates. Delegates were defined as proxies for adults; surrogates were defined as proxies for children or adolescents younger than 18 years.

Thirteen articles described care partner portal use among adolescent (n = 3) or adult (n = 10) patients (Table 2). These analyses were about evenly split between survey-based (n = 6) and electronic medical record–based (n = 7) data. About half were conducted at academic medical centers (n = 6) or integrated managed care consortiums (n = 2). Among adult samples, 2 of 10 articles reported less than 10% of care partners used the portal, 3 of 10 articles reported 10% to 30% of care partners used the portal, and half (5 of 10 articles) reported greater than 30% of care partners used the portal. All 3 articles on adolescents reported greater than 30% of care partners used the portal.

Six articles examined secure messaging content to determine care partner authorship of messages sent through adolescent (n = 1) or adult (n = 5) patient portal accounts (Table 2). Half of these analyses were conducted at academic medical centers or integrated managed care consortiums (n = 3). Two-thirds of articles (n = 4) examined all registered users and 1 of 3 of articles (2 of 6 articles) examined cohorts of specific populations (persons with diabetes and adults aged ≥85 years). The 3 studies that examined care partner messaging with their own identity credentials found these messages comprised less than 3% of adult patient messages. Of the 4 studies examining care partner messaging with patient identity credentials, 1 article reported that care partners authored less than 5% of messages, 1 article reported care partners authored 5% to 20% of messages, and half (2 of 4 articles, which studied persons with diabetes and adults aged ≥85 years) reported that care partners authored 20% to 60% of messages. The 1 study of adolescents reported 20% to 60% of messages were sent by care partners using the adolescent’s identity credentials.

### Factors Associated With Care Partner Use of the Patient Portal

**Figure 2.**
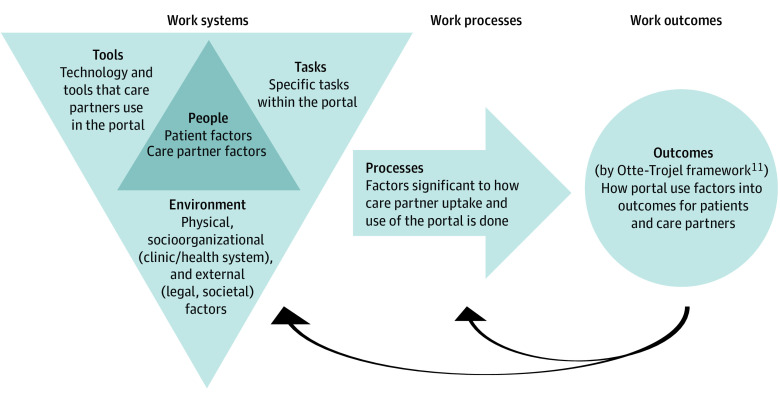
Factors Affecting Care Partner Uptake and Use of the Patient Portal Applied to the Systems Engineering Initiative for Patient Safety Simplified Model

Thirty-two articles reported on factors associated with care partner uptake or use of the portal (Table 3). These factors were categorized by domains of the SEIPS model, differentiating person factors (reporting on patients and care partners separately), environment, tasks, and organization (Figure 2).

**Table 3.** Factors Associated With Care Partner Uptake and Use of the Patient Portal by the SEIPS Model

SEIPS factor	No. of identified studies by study design							Total studies, No.
	Qualitative			Quantitative				
	Facilitator	Barrier	Neither	Facilitator	Barrier	NS	Neither	
**Work system factors (person-patient)**								
Need for assistance with health system navigation	3	1	0	3	0	0	0	7
Illness severity	1	0	1	4	1	0	0	7
Adolescents: younger age	0	0	1	2	0	0	1	4
Adults: older age	0	0	0	3	0	0	1	4
Mental health condition	0	1	0	1	0	0	0	2
White race	0	0	0	0	1	1	0	2
**Work system factors (person-care partner)**								
Health literacy level/educational level	2	0	0	3	0	3	0	8
Female gender	0	0	0	5	0	1	0	6
Relationship to patient: family vs nonfamily	1	0	0	5	0	0	0	6
White race	0	0	0	3	0	2	0	5
Household income	0	0	0	3	1	1	0	5
Technology experience	1	0	0	2	0	0	0	3
**Work system factors: environment (physical, socioorganizational, external)**								
Organizational policy and functionality to enable proxy access	0	1	1	0	1	0	2	5
Clinic staff awareness of proxy access	0	0	0	0	1	0	0	1
State laws	0	0	1	0	0	0	0	1
Internet access	1	0	0	0	0	0	0	1
**Work system factors (tasks)**								
Access to information	5	0	3	3	0	0	1	12
Coordination of care	2	0	0	6	0	0	0	8
**Processes factors: how work is done and how it flows (care processes)**								
Privacy and security	2	2	3	0	0	0	1	8
Review of proxy access status	1	0	2	0	0	0	4	7
Convenience of access type	0	0	1	0	0	0	0	1

Abbreviations: NS, nonsignificant; SEIPS, System Engineering Initiative for Patient Safety.

#### People: Patient Factors

Need for assistance with health system navigation was consistently found to facilitate care partner use of the portal (7 articles)^26,39,40,41,42,43,44^ due to gaps in technology literacy,^44^ English proficiency,^39^ and illness severity.^21,39,45,46,47,48,49^ Younger adolescent age (2 of 4 articles)^20,38^ and older age among adults (3 of 4 articles)^26,39,50^ facilitated care partner use of the patient portal. Findings regarding race and ethnicity were limited and mixed (2 articles).^39,51^

### Care Partner Factors

Greater health literacy and educational level (5 of 8 articles),^25,26,45,52,53^ female gender (5 articles),^25,45,47,48,54^ and being a family member (6 articles)^25,26,42,45,47,53^ were facilitators of care partner use of the portal. Evidence regarding care partner race (5 articles)^25,45,53,54,55^ and household income (5 articles)^21,25,45,53,55^ was mixed. Two articles^26,54^ examined care partners’ use of their own portal and both found it to be a facilitator.

#### Environmental Factors

Organizational factors were most often reported as impeding care partner portal use (3 of 5 articles)^47,52,56^ or spoke to relevance without specifying directionality (3 of 5 articles).^46,57,58^ For example, 1 study^56^ found 7% of hospital personnel did not know about proxy access and nearly half (45%) endorsed patient sharing of credentials with care partners. Two articles^57,58^ describing organizational policies and procedures described the value of graduated proxy access in which the patient controls levels of information access and use of specific functionality. One article^46^ highlighted the importance of state information privacy laws.

#### Tasks

The ability to access patient health information was consistently identified as facilitating care partner portal use (8 of 12 articles),^22,26,42,44,45,59,60,61^ with the remaining 4 articles^49,50,52,62^ emphasizing importance without specifying directionality and identifying care coordination (eg, messaging, appointment scheduling, and filling prescriptions) as facilitating care partner portal use (8 articles).^30,40,42,45,50,52,59,61^

#### Care Processes

Findings relating to care process factors were nuanced and complex. In the 8 articles^20,40,41,44,46,49,60,62^ that reported on privacy and security, 2^40,44^ found privacy and security considerations were a facilitator, 2^46,60^ found them to be a barrier, and 4^20,41,49,62^ did not specify directionality. For example, 2 articles^40,44^ reported that privacy and security facilitated care partners’ portal use by explicitly granting permission. However, 2 articles^46,60^ reported that privacy and security was a perceived barrier to care partner portal use as patients were concerned about another person accessing their personal health and billing information.

Seven articles^20,44,57,58,59,62,63^ reported on health care system processes for proxy access review, although little insight could be gleaned regarding specific practices. In 1 article,^44^ patients voiced that both giving and removing care partner access made them feel in control of health information sharing. In 2 articles,^46,59^ patients expressed the desire to differentiate graduated access to care partners for specific functionality.

### Consequences of Care Partner Use of the Patient Portal by the Otte-Trojel Framework

Thirteen articles,^21,25,41,42,44,45,52,59,61,62,64,65,66^ primarily qualitative (8 of 13 articles),^41,42,44,45,59,63,64,66^ reported on consequences of care partner portal use (eTable 4 in Supplement 1). Care partner use of the portal was found to contribute insight into patient health and personhood ( 9 of 16 articles).^21,41,42,44,59,61,62,64,65^ Care partners were described as an interpreter of health information in the portal,^42,64^ thereby enhancing patient understanding.^41^ Care partner portal access was reported to benefit continuity of care (8 of 16 articles)^21,25,41,44,59,61,65,66^ by improving recall of the plan of care^61^ and understanding reasons for referrals.^65^ Seven articles^42,44,45,61,64,65,66^ reported that care partner portal use affected activation of health information by, for example, promoting desired health behaviors.^44,64^ Six articles^25,42,52,60,61,66^ reported on convenience of care partner use of the patient portal, for example, to “pull-up” the portal when the patient was unavailable^66^ or in the event of an emergency.^42^

## Discussion

To our knowledge, this is the first study to assemble evidence describing the prevalence and consequences of care partner uptake and use of the patient portal and systematically evaluate factors that facilitate or impede this involvement. We found that formal registration for proxy (shared) portal access is low and that care partners more often rely on patient identity credentials. Care partner characteristics, including female gender, family relationship, and greater capacity to engage in health system navigation, were identified as potentially associated with portal use, as was assisting patients with markers of greater vulnerability. Environmental and process factors, including transparency of registration procedures and graduated access type, were identified as important to patients and care partners yet were rarely reported at a health system level. Both patients and care partners reported utility in access to information and coordination of care made possible through the portal, and care partner use of the portal was overwhelmingly associated with positive consequences through mechanisms involving insight into patient health and personhood, activation of information, continuity of care, and convenience.

By compiling available evidence regarding care partner involvement in the patient portal, our study contributes new knowledge in an area that has been only anecdotally recognized to date. The patient portal has been embraced as a mainstream strategy to engage patients in their care,^9,18^ yet care partners have been largely excluded from accumulating knowledge and interventional efforts,^9,18,19,67^ despite their essential role in managing and coordinating care alongside patients who are most vulnerable.^68,69,70^ One small study^21^ suggests the feasibility of systems-level strategies to increase care partner registration and use of portal, although further work is needed to understand prospects for broader scaling. We observed similar issues across the age span from adolescents through older adults, with care partners (from parents of adolescents to adult children of older adults) primarily accessing the patient portal using patient log-in credentials.^51,71^ Both patients and care partners were found to express preferences for improved functionality around proxy access.^46^

Our findings suggest the relevance of organizational factors in care partner uptake of the patient portal but do not indicate specific health system policy, technology, or workflows that facilitate proxy access and instead demonstrate inconsistent policies, features, and awareness surrounding proxy access. Sharing credentials can lead to data security and privacy problems by revealing more information than desired by the patient^30,42,56^ and contribute to confusion and mistakes when clinicians do not know with whom they are interacting electronically^30^ or when legal documents submitted through the patient portal by someone other than the patient must be retracted.^72,73^ Findings from our study suggest the need for stronger evidence regarding best practices in clinician- and electronic medical record vendor–initiated efforts to raise awareness of proxy access and simplify proxy registration. Despite patients wanting greater control over their health information, few health systems afford graduated portal access allowing patients to limit specific portal features available to a care partner; other systems limit patients to 1 registered care partner at a time.^20^

Nearly half of the articles in this review were published between 2020 and 2022, perhaps reflecting increased interest and use of the patient portal during the pandemic.^74,75^ However, we found no temporal difference in care partners’ use of the patient portal in more recent years. Our results suggest that, despite expanded functionality and use of the patient portal, shared and proxy portal registration and use remain limited. Strategies used to increase patient portal access among patients, including targeting both patients and clinicians or staff,^76^ could be adapted to increase proxy access.

Patient portals have been widely scaled throughout care delivery (as many as 90% of health care systems and professionals offer portal access),^77^ yet just 38% of individuals reported they accessed the portal in a national survey conducted in 2020.^78^ For patients who could most benefit from transparent information access and bidirectional communication with clinicians, the patient portal is more often out of reach due to lower health literacy, limited technology access and experience, or cognitive impairment.^19,67,76^ While training could be a valuable step in systems-level strategies that respect patients’ preferences in managing their care,^29^ such efforts are limited in mainstream care.^28^ Our review extends observations supporting the role of care partners as both an underused and underevaluated aspect of patient engagement^76^ by assembling the available but diffuse evidence base regarding their role and its consequences in attenuating patient barriers with health system navigation through patient portal access.

### Limitations

This scoping review has limitations. The review was limited to studies that explicitly examined care partner use of portals. Studies were excluded that examined portal use among both patients and care partners without specifically differentiating between the 2 user groups. Factors influencing patient uptake of the portal would directly affect care partner uptake, but these studies were outside of the scope of this review. Most studies did not identify the relationship of the care partner to the patient. As care partner relationship may affect legal access to the patient’s health information, it is an important consideration that merits interrogation in future studies. Several of the studies in this review were conducted at the same institution and on the same data set or were qualitative studies with rich data but small sample sizes. Thus, there are limitations on our ability to generalize the findings of this review to the larger population. Studies were overwhelmingly observational, we did not appraise the quality of the evidence, and it was not possible to conclusively determine whether and which use of portal functionality (eg, messaging, scheduling, or prescription refills) directly increased care partner uptake.

## Conclusions

The patient portal is increasingly a prerequisite to accessing and coordinating care by enabling asynchronous patient-clinician interaction, facilitating broader access to health information, and affording greater convenience in managing health tasks, such as scheduling appointments and filling prescriptions.^9,11^ This review noted that care partners commonly use the patient portal but that their interactions more often occur informally through sharing of patient identity credentials and that health care systems have to date devoted limited attention toward clarifying and facilitating appropriate involvement. Study findings link to ongoing practice initiatives focused on creating learning health systems,^79,80^ particularly for at-risk subgroups (eg, persons with dementia), and embedded pragmatic trials that leverage health information technology to scale best practices.^81^ Organizations tasked with motivating care quality, such as the Joint Commission and the Office of the National Coordinator for Health Information Technology, must drive transparency and accountability in health systems’ reporting on shared access uptake and ways they promote functionality for user-friendly shared access, particularly for subgroups in which care partners play a large role in care management. For example, electronic health records have, so far, fallen short on serving the specific needs of older adults,^28^ and shared access is highly aligned with such initiatives as the age-friendly health system.^29,82^ This scoping review provides a grounding for the current state of proxy access and suggests the need for greater attention to relevant policy and organizational practices.

## Notes

Supplement 1. **eTable 1.** Search Strategy **eTable 2.** Data Extraction Table **eTable 3.** Detailed Characteristics of Studies Included in Scoping Review **eTable 4.** Consequences of Care Partner Use of the Patient Portal by the Otte-Trojel Framework
eReferences

Supplement 2.
Data Sharing Statement
