# Supplementary material for: Patient Portals to Support Care Partner Engagement in Adolescent and Adult Populations: A Scoping Review
Source: JAMA Netw Open. 2022 Dec 28;5(12):e2248696. doi: 10.1001/jamanetworkopen.2022.48696 (PMC9857556; doi:10.1001/jamanetworkopen.2022.48696)
Supplement: Supplement 1. — eTable 1. Search Strategy eTable 2. Data Extraction Table eTable 3. Detailed Characteristics of Studies Included in Scoping Review eTable 4. Consequences of Care Partner Use of the Patient Portal by the Otte-Trojel Framework eReferences [file jamanetwopen-e2248696-s001.pdf]

Supplementary Online Content

Gleason KT, Peereboom D, Wec A, Wolff JL. Patient portals to support care partner engagement in adolescent and adult populations: a scoping review. *JAMA Netw Open*. 2022;5(12):e2248696. doi:10.1001/jamanetworkopen.2022.48696

eTable 1. Search Strategy

eTable 2. Data Extraction Table

eTable 3. Detailed Characteristics of Studies Included in Scoping Review

eTable 4. Consequences of Care Partner Use of the Patient Portal by the Otte-Trojel Framework

eReferences

This supplementary material has been provided by the authors to give readers additional information about their work.

© 2022 Gleason KT et al. *JAMA Network Open.*

eTable 1. Search Strategy

We applied the following search strategy to PubMed, Web of Science, Embase, and PsychInfo:

**Search Concept**	**Search line number**	**Search strategy**
**Care partner**	1	"Caregivers"[MeSH Terms]
	2	"Caregiver*" ti,ab,kw,kf
	3	"Care Giver" ti,ab,kw,kf
	4	"Spouse Caregiver*" ti,ab,kw,kf
	5	"delegate*" ti,ab,kw,kf
	6	"surrogate*" ti,ab,kw,kf
	7	"parent child relations"[MeSH Terms]
	8	child relationship, parent[MeSH Terms])
	9	child of impaired parents[MeSH Terms
	10	"legal guardians"[MeSH Terms]
	11	"legal guardians/legislation and jurisprudence"[MeSH Terms])
	12	"mother child relations"[MeSH Terms]
	13	"father child relation"[MeSH Terms])
	14	"caregivers/organization and administration"[MeSH Terms]
	15	caregiver, family[MeSH Terms
	16	Caregiver, spouse[MeSH Terms]
	17	or/1-17
**Patient portal**	18	"patient portal*" ti,ab,kw,kf
	19	"patient portal" [MeSH Term]
	20	"Mychart" ti,ab,kw,kf
	21	Or/18-21
**Total**	22	17 and 21

eTable 2. Data Extraction Table

Information to Extract	Definitions and Details	Example Extraction
Extractor Initials	Your initials	K.G
Author	First author of publication	Latulipe
Year	Year of publication	2015
Country	Country of publication	US
Sample Size and Composition	Size of sample and the unit, e.g. patients, care partners, providers, hospitals etc.	51 (36 patients + 16 care partners)
Journal	Publishing journal	Proc SIGCHI Conf Hum Factor Comput Syst
Volume, Issue, Pages	Publication's volume, issue and pages in journal	2859-3868
Title	Title of publication	Design Considerations for Patient Portal Adoption by Low-Income, Older Adults
Data Collection	Method(s) of data collection (EMR data, survey, interview/focus groups, multiple)	Interviews/focus groups
Approach	Qualitative/quantitative/mixed	Qualitative
Patient Age, Population	Patient age group and other population factors	65+, low-income patients and caregivers
Setting	Study site/location description	3 health systems
Rationale for Inclusion	Select research question(s) that apply: RQ1, RQ2, RQ3	RQ2, RQ3
Provider	Name of healthcare delivery organization, if applicable	Kaiser Permanente, Northern California
Inclusion Criteria	The study's inclusion criteria for the sample	Patient with 1+ chronic condition, from clinical registry
EMR Vendor	EMR vendor, if appliable	Epic
Patient Uptake	Patient uptake of the patient portal (n, %), as reported in the study	76.30%
Proxy Uptake	Proxy uptake of the proxy portal (n, %), as reported in the study	17.60%
Messages Sent by Patient	Messages sent by patient via patient portal (n, %), as reported in the study	904 of 1000 (90.4%) initiated by patient; 1445 of 2715 messages (53.2%) sent by patient
Messages Sent by Care Partner	Messages sent by care partner via patient portal (n, %), as reported in the study	41 of 1000 threads (4.1%) initiated by care partner; 69 of 2715 messages (2.5%) sent by care partner

© 2022 Gleason KT et al. *JAMA Network Open.*

Factors affecting care partner uptake and use of the patient portal by the SEIPS Model(1, 2)		
**Work System Factors (Person - Patient)**		
Age	Data indicating patient age as a factor affecting care partner uptake/use of patient portal	"There was no difference in mean age between patients who had messages apparently sent by proxies through patient portal accounts versus proxy portal accounts."
Gender	Data indicating patient gender as a factor affecting care partner uptake/use of patient portal	"Patients who had proxies send messages through patient accounts were more likely to be married and male compared with patients whose proxies used proxy accounts to send message."
Need for asisstance	Data indicating patient's need for assistance (english proficiency, comfort with technology) as a factor affecting care partner uptake/use of patient portal	"Patients with a higher % of proxy-sent messages had higher rates of limited English proficiency (16.1% vs 3.2% vs 3.5%, p<0.05)"
Illness severity	Data indicating patient illness severity as a factor affecting care partner uptake/use of patient portal	"Caring for a patient with a chronic condition was associated with greater odds of portal use for caregiving (OR: 1.66, P = 0.022)."
Mental Health condition	Data indicating patient's mental health condition as a factor affecting care partner uptake/use of patient portal	"Caring for a patient with a mental health condition was associated with greater odds of portal use for caregiving (OR: 1.71, P = 0.013)."
Race	Data indicating patient race as a factor affecting care partner uptake/use of patient portal	"patients predicted as having high percent PPSMs [predicted proxy secure messaging] were … more likely to be … non-white (80.7% vs 66.8% vs 72.4%, p < 0.05)"
**Work System Factors (Person - Care Partner)**		
Gender	Data indicating care partner's gender as a factor affecting portal uptake/use	"Being female (OR 2.58, 95% CI 1.40-4.75; P=.002) was associated with a significantly higher likelihood of using their care recipient's portal"
Race	Data indicating care partner's race as a factor affecting portal uptake/use	"care recipient portals were more likely to be accessed by White caregivers"
Relationship to patient	Data indicating care partner's relationship to patient as a factor affecting care partner uptake/use of the patient portal	"caregivers supporting a parent were significantly less likely than those supporting a spouse/partner to report using the online medical record (OR = 0.28, p = 0.01)."

© 2022 Gleason KT et al. *JAMA Network Open.*

Health literacy level/ education	Data indicating care partner's health literacy level/ education level as a factor affecting care partner uptake/use of the patient portal	"for most tasks, a higher percentage of patients than caregivers required assistance from staff in order to complete the task… Explaining medical terms was the most commonly cited wish."
Technology experience	Data indicating care partner's technology experience as a factor affecting care partner uptake/use of the patient portal	"Care partners were more likely than patients to report using the Internet daily (87.6% versus 55.0%, P<.001) and using a computer to perform health management activities (95.5% and 48.4%, P<.001)"
Household income	Data indicating care partner's household income as a factor affecting care partner uptake/use of the patient portal	"Care partners who logged into MyChart were more likely to… have worked in the last week (70.0% vs 42.4%; p=0.03) than care partners who did not."
Illness/ Illness severity	Data indicating care partner's illness/ illness severity as a factor affecting care partner uptake/use of the patient portal	"Having a chronic health condition was associated with greater odds of portal use for self-care (OR: 1.33, P < 0.001) but not for caregiving."
Mental Health condition	Data indicating care partner's mental health condition as a factor affecting care partner uptake/use of the patient portal	"Having a mental health condition was associated with greater odds of portal use for self-care (OR: 1.23, P = 0.01) but reduced odds of portal use for caregiving (OR: 0.61, P = 0.03)."
**Work System Factors - Environment (Physical, Socio-organizational, External)**		
Clinical-site factors	Data indicating factors at the clinical site (organizational, technical or policy, or lack of clinician/staff awareness of proxy access) limiting availability of proxy access	"7% of personnel did not know about proxy accounts, 45% endorsed password-sharing for caregivers to the primary portal account"
Location/setting	Data indicating location or setting (of healthcare delivery or of patient/care partner use of the portal) as a factor affecting care partner use/uptake of the patient portal	68% of hospitals offered proxy accounts to caregivers
Internet access	Data indicating care partner's internet access as a factor affecting care partner use/uptake of the patient portal	internet access is less available in rural settings, variable in low-income urban areas

© 2022 Gleason KT et al. *JAMA Network Open.*

**Work System Factors (Tasks)**		
Access to information	Data indicating information types accessed by care partners (lab results, visit notes, patient health status details) as a factor affecting care partner uptake/use of the patient portal	"Care partners read visit notes more frequently than patients. 98% or care partners indicated that 'making visit notes avilable on the electronic portal was a good idea.' "
Coordination of care	Data indicating patient portal functionality accessed by care partners (messaging, appointment scheduling, filling prescriptions) as a factor affecting care partner uptake/use of the patient portal	"Patients indicated that they shared access to their patient portal account to facilitate involvement of care partners in managing healthcare activities (41.9%)"
**Processes factors (care processes): how work is done and how it flows**		
Privacy and security	Data indicating that privacy and security as a factor affecting care partner uptake/use of the patient portal	68.1 % of respondents indicated no concerns about privacy and confidentiality
Review of proxy access status	Data indicating the patient's ability to control proxy access as a factor affecting care partner uptake/use of the patient portal	Patients desired graduated access rights so patients could determine more limited access in some cases; caregivers were in favor of broader access
Convenience of access type	Data indicating ease in establishing proxy access as factor affecting care partner uptake/use of the patient portal	Care partners preferred accessing the patient portal through the patient's account because it was more convenient than proxy
**Consequences of Care Partner Use of the Patient Portal by the Otte-Trojel Framework(3)**		
Insight into Patient Health and Personhood	Data indicating that care partner access to patient information through the patient portal enables or facilitates patients and care partners to be involved in its application and in ensuring its accuracy and comprehensiveness.	"One of the main reasons participants felt caregiver access was beneficial was in the caregiver's ability to help them understand the information on the portal, such as doctor's messages and test results."
Activation of Information	Data indicating that care partner use of the patient portal increases the effectiveness and targetability of information	"Among those using the portal as a care partner, 94.2% reported that it was more convenient than other ways of participating in another person's health care."
Continuity of Care	Data indicating that care partner use of the patient portal was relevant to continuity of care	Care partners who accessed the portal reported a higher ability to remember the patient's care plan (94.3% and 93.5%), felt more in control of care (88.6% and 89.7%).

© 2022 Gleason KT et al. *JAMA Network Open.*

Convenience	Data indicating that care partner use of the patient portal facilitates addressing health care needs	"Several patients discussed the benefits of caregivers being able to access the portal in case of an emergency."

© 2022 Gleason KT et al. *JAMA Network Open.*

eTable 3. Detailed characteristics of studies included in scoping review

First Author, Year	Journal	Title	Sample	Patient age category	Sample characteristics	Data collection	Approach
Barron, 2014	Stud Health Technol Inform	Exploring three perspectives on feasibility of a patient portal for older adult	7 patients, 16 care partners	Older adults	Illness-specific	Focus groups/interviews	Qualitative
Bergman, 2008	Perspect Health Inf Manag	Teen use of a patient portal: a qualitative study of parent and teen attitudes	35 patients, 34 care partners[a]	Adolescents	General	Focus groups/interviews	Qualitative
Chimowitz, 2018	Jt Comm J Qual Patient Saf	Empowering informal caregivers with health information: OpenNotes as a safety strategy	6,908 patients, 150 care partners[b]	Adults	General	Multiple	Quantitative
Crotty, 2015	JAMA Intern Med	Information sharing preferences of older patients and their families	30 patients, 23 care partners	Older adults	General	Focus groups/interviews	Qualitative
Davis, 2012	Electron. J. Health Inform	Improving informal caregiver engagement with a patient web portal	343 care partners	Adults	General	Survey	Mixed
Dickman Portz, 2021	J Palliat Med	Characteristics of patients and proxy caregivers using patient portals in the setting of serious illness and end of life	12,646 patients, 311 care partners	Adults	Illness-specific	EMR/ patient portal data	Quantitative
Fitzsimons, 2021	Epilepsy Behav	Democratizing epilepsy care: utility and usability of an electronic patient portal	72 patients, 18 care partners	Adults	Illness-specific	Survey	Quantitative
Gupta, 2021	JMIR Cancer	Electronic health record portal use by family caregivers of patients undergoing hematopoietic cell transplantation: United States national survey study	948 care partners	Adults	Illness-specific	Survey	Quantitative
Hodgson, 2022	J Patient Exp	Utilization of EHR to improve support person engagement in health care for patients with chronic conditions	12 patients, 7 care partners, 2 providers	Adults	Illness-specific	Focus groups/interviews	Qualitative
Iott, 2020	J Gen Intern Med	Family caregiver access of online medical records: findings from the Health Information National Trends Survey	191 care partners	Adults	General	Survey	Quantitative
Ip, 2021	JAMA Netw Open	Assessment of prevalence of adolescent patient portal account access by guardians	3,429 patient portal accounts	Adolescents	General	EMR/ patient portal data	Quantitative

© 2022 Gleason KT et al. *JAMA Network Open.*

Jackson, 2021	Patient Educ Couns	Care partners reading patients' visit notes via patient portals: characteristics and perceptions	28,782 patients, 874 care partners	Adults	General	Multiple	Mixed
Latulipe, 2015	Proc SIGCHI Conf Hum Factor Comput Syst	Design considerations for patient portal adoption by low-income, older adults	36 patients, 16 care partners	Older adults	General	Focus groups/inter views	Qualitative
Latulipe, 2018	J Med Internet Res	Insights into older adult patient concerns around the caregiver proxy portal use: qualitative interview study	10 patients	Older adults	Illness-specific	Focus groups/inter views	Qualitative
Latulipe, 2020	JAMA Intern Med	Security and privacy risks associated with adult patient portal accounts in US hospitals	102 hospitals	NA or unknown	General	Survey	Quantitative
Mayberry, 2011	Diabetes Technol Ther	Bridging the digital divide in diabetes: family support and implications for health literacy	61 patients	Adults	Illness-specific	Multiple	Mixed
Nippak, 2015	J Hosp Admin	Family attitudes towards an electronic personal health record in a long term care facility	65 care partners	NA or unknown	Illness-specific	Survey	Mixed
Osborn[c], 2011	J Am Med Inform Assoc	MyHealthAtVanderbilt: policies and procedures governing patient portal functionality	>129,800 registered users	All ages	General	EMR/ patient portal data	Mixed
Parsons, 2020	J Am Med Inform Assoc	Preserving privacy for pediatric pateint and families: use of confidential note types in prediatric ambulatory care	402, 964 clinic notes	Adolescents	General	EMR/ patient portal data	Quantitative
Pecina, 2020	Telemed J E Health	Frequency of and factors associated with care partner proxy interaction with health care teams using patient portal accounts	3,000 messages	Adults	General	EMR/ patient portal data	Quantitative
Raj, 2021	JMIR Aging	Evaluation of family caregivers' use of their adult care recipient's patient portal from the 2019 Health Information National Trends Survey: secondary analysis	320 care partners	Adults	General	Survey	Quantitative
Ramirez-Zohfeld, 2020	J Am Geriatr Soc	Use of electronic health records by older adults, 85 years and older, and their caregivers	62 patients, 82 care partners[d]	Older adults	General	EMR/ patient portal data	Mixed
Reed, 2018	JAMA Intern Med	Communicating through a patient portal to engage family care partners	1,824 patients/care partners[e]	Adults	Illness-specific	Survey	Mixed

© 2022 Gleason KT et al. *JAMA Network Open.*

Schnock, 2019	J Med Internet Res	Acute care patient portal intervention: portal use and patient activation.	2,974 patients	Adults	Illness-specific	Multiple	Quantitative
Semere, 2019	J Gen Intern Med	Secure messaging with physicians by proxies for patients with diabetes: findings from the ECLIPPSE study	9,856 patients	Adults	Illness-specific	EMR/ patient portal data	Quantitative
Sharko, 2018	J Am Med Inform Assoc	Variability in adolescent portal privacy features: how the unique privacy needs of the adolescent patient create a complex decision-making process	25 health systems	Adolescents	General	Focus groups/inter views	Qualitative
Shimada, 2017	J Am Med Inform Assoc	An analysis of patient-provider secure messaging at two Veterans Health Administration medical centers: message content and resolution through secure messaging	1,000 message threads, 2,715 messages	Adults	General	EMR/ patient portal data	Quantitative
Steitz, 2017	Appl Clin Inform	Long-term patterns of patient portal use for pediatric patients at an academic medical center	17,128 patients	Adolescents	General	EMR/ patient portal data	Quantitative
Steitz, 2019	JAMIA Open	Policies and procedures governing patient portal use at an Academic Medical Center	375,517 registered accounts for 402,744 patients	All ages	General	EMR/ patient portal data	Quantitative
Strudwick, 2020	Inform Health Soc Care	Identifying indicators of meaningful patient portal use by psychiatric populations.	12 patients, 6 care partners, 5 providers[f]	All ages	Illness-specific	Focus groups/inter views	Qualitative
Szilagyi, 2020	J Am Med Inform Assoc	Pediatric patient portal use in one health system	39,871 patients	Adolescents	General	EMR/ patient portal data	Quantitative
Tieu, 2015	J Med Internet Res	Barriers and facilitators to online portal use among patients and caregivers in a safety net health care system: a qualitative study	11 patients, 5 care partners	Adults	Illness-specific	Focus groups/inter views	Qualitative
Turner, 2021	JAMA Netw Open	Use of electronic health record patient portal accounts among patients with smartphone-only internet access	4,200 patients, 612 care partners	Adults	General	Survey	Quantitative
Weis, 2020	BMC Med Inform Decis Mak	Caregivers' role in using a personal electronic health record: a qualitative study of cancer patients and caregivers in Germany	22 patients, 9 care partners	Adults	Illness-specific	Focus groups/inter views	Qualitative

© 2022 Gleason KT et al. *JAMA Network Open.*

Wolff, 2016	J Am Med Inform Assoc	Patients, care partners, and shared access to the patient portal: online practices at an integrated health system	323 patients, 389 care partners	Adults	General	EMR/ patient portal data	Quantitative
Wolff, 2018	J Am Med Inform Assoc	An environmental scan of shared access to patient portals	20 health systems	NA or unknown	General	Survey	Quantitative
Wolff, 2021	NPJ Breast Cancer	A randomized intervention involving family to improve communication in breast cancer care	118 patient/care partner dyads	Adults	Illness-specific	Multiple	Quantitative
Wolff, 2017	J Am Med Inform Assoc	Inviting patients and care partners to read doctors' notes: OpenNotes and shared access to electronic medical records	184 patients, 252 care partners	Adults	General	Multiple	Quantitative
Wolff, 2019	Breast Cancer Res Treat	Sharing in care: engaging care partners in the care and communication of breast cancer patients.	132 patient/care partner dyads	Adults	Illness-specific	Multiple	Quantitative
Xie, 2021	J Adolesc Health	Ensuring adolescent patient portal confidentiality in the age of the Cures Act Final Rule	3,701 patient portal accounts	Adolescents	General	EMR/ patient portal data	Quantitative
Zupa, 2021	J Gen Intern Med	Impact of a dyadic intervention on family supporter involvement in helping adults manage Type 2 Diabetes	239 patient/care partner dyads	Adults	Illness-specific	Survey	Quantitative

Footnotes:
[a]The study team calculated number of patients and care partners from available data.
[b]This sample included 19 parents and 17 teens who participated in focus groups and 15 parents and 18 teens who provided feedback via online bulletin boards.
[c]The breakdown of patients vs. care partner users was not provided.
[d]This study reviewed 1254 portal messages from patients and care partners.
[e]About 76 percent of the sample used the patient portal for their own care needs and of this group, 27.5 percent also used the portal as a care partner.
[f]The providers in this study were peer support workers. The patients were referred to as consumers of psychiatric services.

© 2022 Gleason KT et al. *JAMA Network Open.*

eTable 4. Consequences of Care Partner Use of the Patient Portal by the Otte-Trojel framework

	Number of Studies			Exemplar quote
	Qualitative	Quantitative	Total studies	
Insight into Patient Health and Personhood	6	3	9	"Caregivers also noted the importance of their role as interpreters of health information: '*I think he would be looking at [the portal] with me but he just doesn't understand so I would just have to relay the message*.' - Female caregiver for parent." Tieu, 2015(4)
Activation of Information	5	2	7	(Care partner) "*We can be better at keeping all our other doctors on the same page as our lead doctor. We form better questions for our next office visits, and we keep the doctor informed with earlier feedback when medications or health conditions change*." Jackson, 2021(5)
Continuity of Care	5	3	8	"*Regardless of the level of support provided, proxies reported EHR access allowed them to provide better support." "They (patients and proxies) highlighted how proxy access assisted with: (a) communicating with the healthcare team, (b) reviewing lab results, (c) scheduling appointments, and (d) filling and picking up mediation refills*." Hodgson, 2022(6)
Convenience	4	2	6	"*Another participant explained how the delegate function would help him when he is traveling and needs care …If I ever end up in an Urgent Care or in an emergency, I now have access to something very quickly that my wife can pull up and [a provider] who doesn't know me can say 'here's what's going on, here are all the medications and the history*.'" Mayberry, 2011(7)

© 2022 Gleason KT et al. *JAMA Network Open.*

eReferences

1. Carayon P, Wooldridge A, Hoonakker P, Hundt AS, Kelly MM. SEIPS 3.0: Human-centered design of the patient journey for patient safety. Appl Ergon. 2020;84:103033.
2. Holden RJ, Carayon P. SEIPS 101 and seven simple SEIPS tools. BMJ Qual Saf. 2021;30(11):901-10.
3. Otte-Trojel T, Rundall TG, de Bont A, van de Klundert J, Reed ME. The organizational dynamics enabling patient portal impacts upon organizational performance and patient health: a qualitative study of Kaiser Permanente. BMC Health Serv Res. 2015;15:559.
4. Tieu L, Sarkar U, Schillinger D, Ralston JD, Ratanawongsa N, Pasick R, et al. Barriers and Facilitators to Online Portal Use Among Patients and Caregivers in a Safety Net Health Care System: A Qualitative Study. J Med Internet Res. 2015;17(12):e275.
5. Jackson SL, Shucard H, Liao JM, Bell SK, Fossa A, Payne TH, et al. Care partners reading patients' visit notes via patient portals: Characteristics and perceptions. Patient Educ Couns. 2022;105(2):290-6.
6. Hodgson J, Welch M, Tucker E, Forbes T, Pye J. Utilization of EHR to Improve Support Person Engagement in Health Care for Patients With Chronic Conditions. J Patient Exp. 2022;9:23743735221077528.
7. Mayberry LS, Kripalani S, Rothman RL, Osborn CY. Bridging the digital divide in diabetes: family support and implications for health literacy. Diabetes Technol Ther. 2011;13(10):1005-12.

© 2022 Gleason KT et al. *JAMA Network Open.*
