# Supplementary material for: Patient Portals to Support Care Partner Engagement in Adolescent and Adult Populations: A Scoping Review
Source: JAMA Netw Open. 2022 Dec 28;5(12):e2248696. doi: 10.1001/jamanetworkopen.2022.48696 (PMC9857556; doi:10.1001/jamanetworkopen.2022.48696)
Supplement: Supplement 2. — Data Sharing Statement [file jamanetwopen-e2248696-s002.pdf]

Data Sharing Statement

Gleason. Patient Portals to Support Care Partner Engagement in Adolescent and Adult Populations. *JAMA Netw Open*. Published December 28, 2022. doi:10.1001/jamanetworkopen.2022.48696

Data
Data available: No

Additional Information
Explanation for why data not available: All data collected are already available in paper, given the nature of a scoping review.
